# The Phosphorylation of PDX-1 by Protein Kinase CK2 Is Crucial for Its Stability

**DOI:** 10.3390/ph10010002

**Published:** 2016-12-28

**Authors:** Sabrina Klein, Rui Meng, Mathias Montenarh, Claudia Götz

**Affiliations:** 1 Medical Biochemistry and Molecular Biology, Saarland University, 66424 Homburg, Germany; klein.sabrina@outlook.de (S.K.); mengruivip@163.com (R.M.); mathias.montenarh@uks.eu (M.M.); 2 Cancer Center of Union Hospital, Tongji Medical College, Huazhong University of Science and Technology, No. 156 Wujiadun, Hankou, Wuhan 430045, China

**Keywords:** pancreatic and duodenal homeobox protein PDX-1, PDX-1 C-terminus interacting factor PCIF1, protein kinase CK2, protein stability

## Abstract

The homeodomain protein PDX-1 is a critical regulator of pancreatic development and insulin production in pancreatic β-cells. We have recently shown that PDX-1 is a substrate of protein kinase CK2; a multifunctional protein kinase which is implicated in the regulation of various cellular aspects, such as differentiation, proliferation, and survival. The CK2 phosphorylation site of PDX-1 is located within the binding region of the E3 ubiquitin ligase adaptor protein PCIF1. To study the interaction between PDX-1 and PCIF1 we used immunofluorescence analysis, co-immunoprecipitation, GST-pull-down studies, and proximity ligation assay (PLA). For the analysis of the stability of PDX-1 we performed a cycloheximide chase. We used PDX-1 in its wild-type form as well as phosphomutants of the CK2 phosphorylation site. In pancreatic β-cells PDX-1 binds to PCIF1. The phosphorylation of PDX-1 by CK2 increases the ratio of PCIF1 bound to PDX-1. The stability of PDX-1 is extended in the absence of CK2 phosphorylation. Our results identified protein kinase CK2 as new important modulator of the stability of PDX-1.

## 1. Introduction

Protein kinase CK2 is a multifunctional and pleiotropic protein kinase that plays critical roles in cell differentiation, proliferation, and survival [1,2,3]. CK2 is a multi-subunit protein kinase which is generated by the association of two catalytic α- and/or α’- subunits (38–42 kDa) with a dimer of the 27 kDa non-catalytic β-subunit. Up to now CK2 is known to phosphorylate more than 400 different proteins and the number is rapidly increasing [4]. CK2 phosphorylation of proteins can modulate the subcellular localization, change DNA binding and transcription factor activities, to mention but a few [4]. There are several reports that CK2 also affects the stability or break-down of proteins [5,6,7,8,9,10].

Recently, we identified the transcription factor PDX-1 as a new substrate for protein kinase CK2 [11]. The pancreatic duodenal homeobox 1 protein (PDX-1), also known as IPF-1, IDX-1, STF-1, GSF, and IUF-1, is expressed in the region of the developing foregut that later becomes the duodenum, distal stomach, and pancreas [12,13]. As development proceeds, PDX-1 expression gets restricted to mostly the hormone-producing β and δ cells of the endocrine pancreas. Murine PDX-1 is a 284 amino acid-long protein with a central homeodomain flanked by proline-rich N- and C-terminal domains. A transactivation domain maps to the N-terminus. The homeodomain contains the DNA binding domain as well as a nuclear localization signal [14,15]. Although the function of the C-terminus is poorly understood, there is some indication that the C-terminus has an inhibitory function whereas other data showed, that the C-terminus is required for full transactivation function [16,17,18]. PDX-1 is a phosphoprotein and a number of different kinases is known to phosphorylate PDX-1 (for review see [19]). Recently, we identified two phosphorylation sites for protein kinase CK2 in the C-terminus of PDX-1, namely threonine 231 and serine 232 [11] and found that the transcription factor activity of PDX-1 is modulated by the CK2 phosphorylation. Moreover, both phosphorylation sites are in the center of the binding domain for PCIF1 (PDX-1 C terminus interacting factor 1) [20], a protein which targets PDX-1 for ubiquitination and proteasomal degradation [21]. PCIF1, or the human homologue SPOP (Speckle type POZ (pox virus and zinc finger protein)), acts as E3 ubiquitin ligase adaptor which binds the Cullin moiety of the E3 ubiquitin ligase complex to their unique substrate binding adaptor proteins [22]. In the present study, we have analyzed whether the stability of PDX-1 and its interaction with PCIF1 might be affected by the phosphorylation of CK2 within the PCIF1 binding region.

## 2. Results

We have previously shown that PDX-1 is a substrate for protein kinase CK2 [11]. Phosphorylation sites were identified as threonine 231 and serine 232 of mouse PDX-1. We further showed that the transcription factor activity of PDX-1 is modulated by CK2 phosphorylation. Since the CK2 phosphorylation site of PDX-1 lies in the middle of the interaction domain with the E3 ubiquitin ligase adaptor protein PCIF1 we wanted to know whether the interaction with and the stability of PDX-1 might be affected by CK2 phosphorylation.

**Figure 1:**
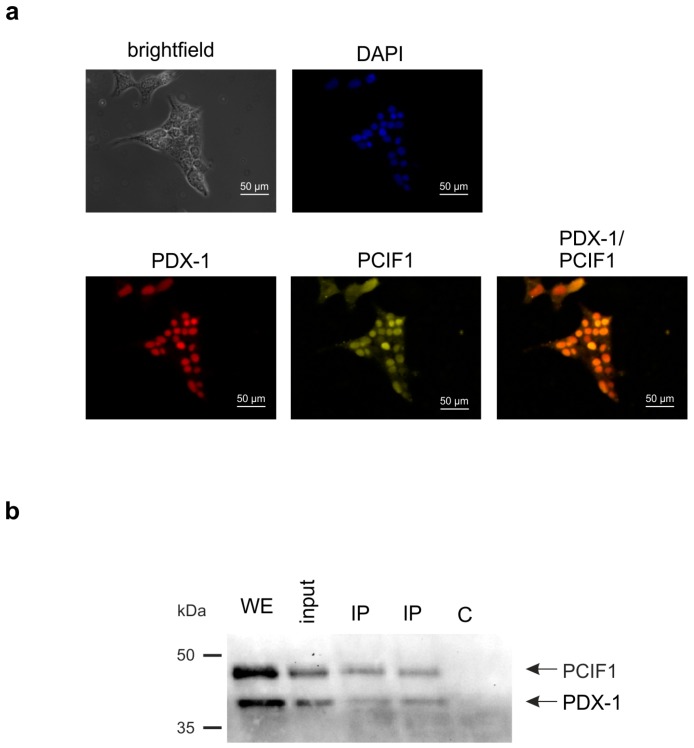
PDX-1 and PCIF1 interaction in MIN6 cells. (**a**) Immunofluorescence analysis of endogenous PDX-1 and PCIF1. MIN6 cells were cultured on coverslips and stained with a polyclonal rabbit antiserum against PDX-1, a goat polyclonal antiserum against PCIF1 (SPOP K-13) and DAPI for nuclei staining. As secondary antibodies, ALEXA-Fluor^TM^488 or ALEXA-Fluor^TM^594 were used. Immunofluorescence was analyzed using a Zeiss Axioskop fluorescence microscope (Zeiss, Jena, Germany). Magnification: 400×; and (**b**) co-immunoprecipitation of PDX-1 and PCIF1 from transfected MIN6 cell extracts. MIN6 cells were transfected with PDX-1 and FLAG-tagged PCIF1 for 24 h. Two mg of cell extract were precleared twice with a mixture of protein A sepharose beads and CL 4-B agarose beads over a period of 1 h. The supernatants were incubated with a rabbit PDX-1 antibody for 2 h. The immunoprecipitated proteins were separated by 12.5% SDS polyacrylamide gel electrophoresis, transferred to a polyvinylidenfluoride (PVDF) membrane and analyzed by Western blot with the mouse monoclonal M2 antibody against the FLAG-tag of PCIF1 and the polyclonal rabbit antiserum against PDX-1. WE: whole cell extract (20 µg), input: 10% of cell extracts used for immunoprecipitation, C: control without antibody, IP: immunoprecipitate, two independent precipitations.

A prerequisite for a physical interaction is the co-localization of two proteins. In a first experiment we performed an in situ immunofluorescence analysis with PDX-1 and PCIF1 specific antibodies in mouse pancreatic MIN6 β-cells. Figure 1a shows the localization of PDX-1 (red) and the localization of PCIF1 (green). Merging both images (yellow color) revealed that PDX-1 and PCIF1 colocalize mostly in the nucleus. The fact that both proteins are present in the same place does not necessarily mean that they interact. In a further experiment we, therefore, performed a co-immunoprecipitation with a PDX-1 specific antibody. As there is a lower abundance of PCIF1 in β insulinoma cells ([20], and our observation), we transfected MIN6 cells with eukaryotic expression constructs for PDX-1 and PCIF1. After transfection, cell extracts were incubated with a PDX-1 specific antibody. Proteins of the immunocomplex were separated by SDS polyacrylamide gel electrophoresis and analyzed by immunoblot analysis with a PDX-1 specific and a FLAG-tag antibody against PCIF1. The result shown in Figure 1b demonstrates that both proteins were immunoprecipitated with the PDX-1 specific antibody (lane IP).

**Figure 2:**
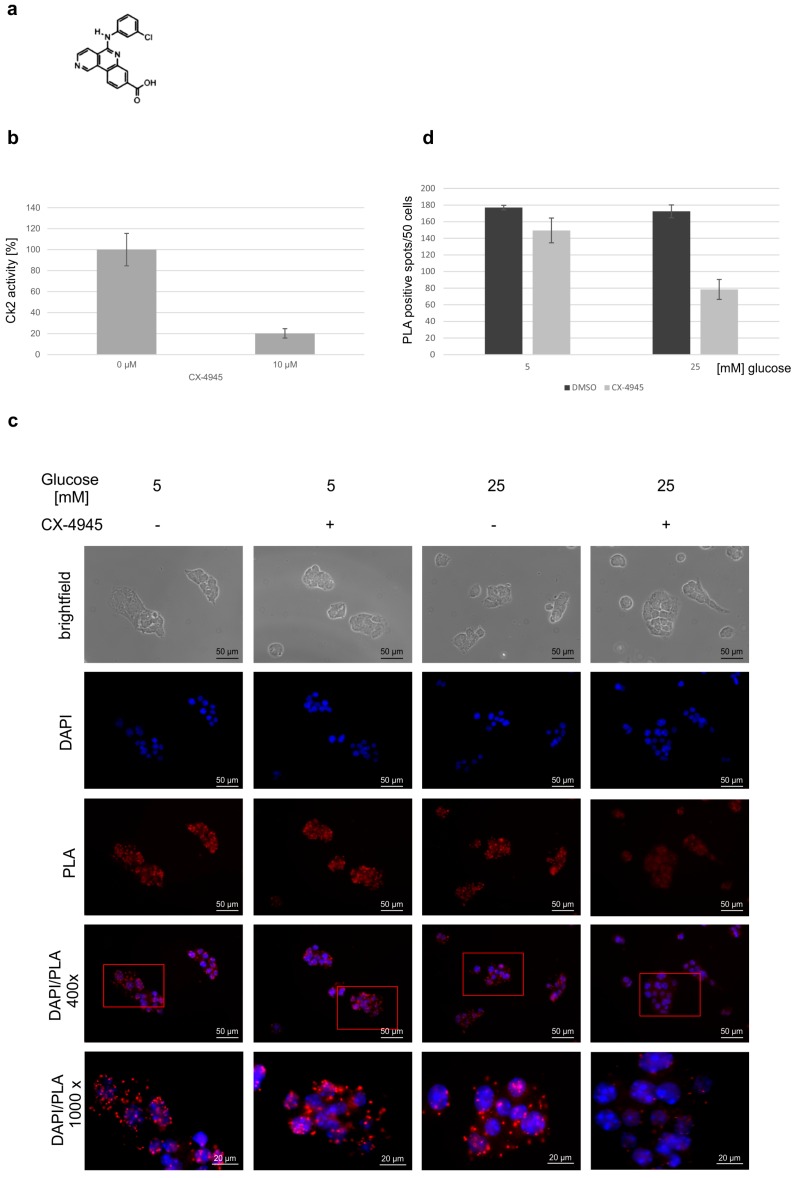
Influence of glucose and CK2 inhibitor CX-4945 on the interaction of PDX-1 and PCIF1. (**a**) Chemical structure of CX-4945 (Silmitasertib); (**b**) in vitro phosphorylation assay in the absence and presence of CX-4945. MIN6 were treated with DMSO as control (0 µM) or with 10 µM CX-4945 for 24 h. Kinase activity of CK2 in the cell extracts was measured with the synthetic CK2 specific peptide substrate, RRRDDDSDDD, in the presence of [^32^P]γATP. Results from three individual experiments are shown; the activity in the control cells was set to 100%; (**c**) Duolink^®^ in situ proximity ligation assay (PLA) of PDX-1 and PCIF1 in MIN6 cells. MIN6 cells were incubated with 5 mM or 25 mM glucose for 4 h after 5 h starvation and simultaneously with 10 µM CX-4945 (+) or an equal volume DMSO for control (−). Cells were subjected to Duolink^®^ in situ proximity ligation assay using antibodies against PDX-1 (polyclonal rabbit antiserum) and PCIF1 (mouse monoclonal antibody SPOP B-8). Immunofluorescence was analyzed using a Zeiss Axioskop fluorescence microscope (Zeiss, Jena, Germany); and (**d**) quantification of the interaction. Single dots were counted in 50 cells of three different areas. Experiments in the absence of CX-4945 were used as reference (100%). The bar graph shows the median results of three independent counts.

A more refined type of analysis to study the interaction of proteins in situ, is the proximity ligation assay PLA [23]. To analyze the role of a CK2 phosphorylation of PDX-1 we inhibited the CK2 activity with the at present most specific CK2 inhibitor CX-4945 [24]. By incubating MIN6 cells for four hours in the presence of 10 µM CX-4945, CK2 activity was reduced to 20% of the activity of untreated cells (−) (Figure 2b). As the transcription factor activity of PDX-1 is glucose responsive [25] and as the interaction with PCIF1 might also be affected by glucose we used glucose concentration of 5 and 25 mM. MIN6 cells were seeded on coverslips, glucose was withdrawn from the medium for 5 h and then added again in a concentration of 5 mM (normoglucose) or 25 mM (high glucose) to stimulate glucose-dependent processes. MIN6 cells were subjected to a PLA analysis with a PDX-1 and a PCIF1 specific antibody. A positive interaction was visualized by red fluorescent dots (Figure 2c). Counting the dots in three different independent areas demonstrated that glucose had no influence on the interaction (average 177 vs. 173 dots/50 cells). However, when cells were treated with the CK2 inhibitor, the mean value decreased to 150 dots/50 cells (85%) under normoglucose and even to 79 dots/50 cells (45%) under high glucose (Figure 2d). Thus, the interaction of both proteins is significantly reduced in the presence of the CK2 inhibitor; the reduction is most obvious under high glucose conditions.

**Figure 3:**
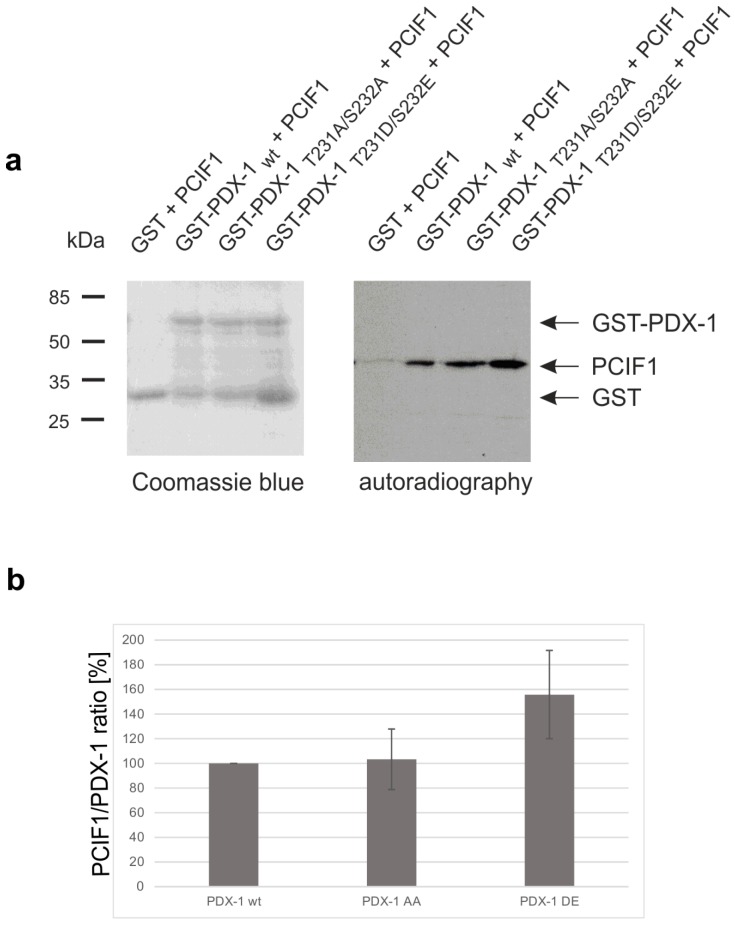
Interaction of PDX-1 and PCIF1 as a function of CK2 phosphorylation. (**a**) GST pull-down analysis of the PCIF1/PDX-1 interaction. About 10 µg GST-PDX-1_WT_, GST-PDX-1_T231A/S232A_, or GST-PDX-1_T231D/S232E_ were incubated with 7.5 µL of in vitro translated and [^35^S]-methionine labeled PCIF1 protein. The formed complex was coupled to GSH sepharose. Proteins eluted from the affinity resins were analyzed on a 12.5% SDS polyacrylamide gel, stained with Coomassie blue and afterwards subjected to autoradiography; and (**b**) after densitometric analysis of the PDX-1 and PCIF1 signals the PCIF1/PDX-1 ratio was calculated and the relative values were presented as bar graphs referred to the interaction of wild-type PDX-1 and PCIF1.

To prove that the interaction of both proteins is phosphorylation-sensitive we performed a GST-pull-down assay with wild-type and phosphomutant proteins [26]. PCIF1 is not a known CK2 substrate; by in silico scanning of the polypeptide chain no sequence with the known consensus motif for CK2 (S/TxxD/E [27]) was found. Thus, we used in vitro translated [^35^S]-methionine labeled PCIF1 in its wild-type form and incubated it with GST-tagged PDX-1 wild-type and the CK2-phosphodeficient (PDX-1_T231A/S232A_) or the CK2-phosphomimicking (PDX-1_T231D/S232E_) mutant. GST-tagged PDX-1 and bound PCIF1 were pulled-down with GSH sepharose, complexes were eluted and separated on an SDS polyacrylamide gel. Proteins were stained with Coomassie blue and subjected to autoradiography. The result is shown in Figure 3a. The Coomassie blue staining demonstrated that we used equal amounts of GST-PDX-1 forms. All PDX-1 variants bound to PCIF1, however, the phospho-mimicking mutant PDX-1_T231D/S232E_ bound considerably more PCIF1. After evaluation of four independent experiments the binding capacity of the D/E mutant was calculated to be 1.5 fold better than for wild-type or the AA mutant (Figure 3b). Thus, we conclude that the CK2 phosphorylated form of PDX-1 binds better to the E3 ubiquitin ligase adaptor protein PCIF1.

**Figure 4:**
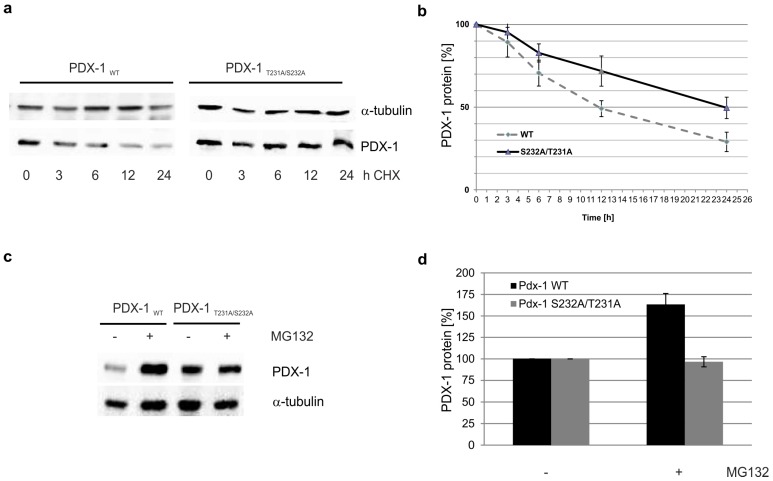
Stability of PDX-1_WT_ and PDX-1_T231A/S232A_ in MIN6 cells. (**a**) MIN6 cells were transfected with FLAG-PDX-1_WT_ or the double mutant FLAG-PDX-1_T231A/S232A_. Thirty hours post transfection, cells were treated with 100 μg/ml cycloheximide (CHX) to inhibit the protein synthesis. Cells were either extracted immediately (0 h) or after 3, 6, 12, and 24 h of treatment. The cell extract was analyzed on a 12.5% SDS polyacrylamide gel and transferred to a PVDF membrane followed by Western blotting with anti-FLAG tag- and anti-α-tubulin- antibodies. α-tubulin was used as a loading control. One representative blot out of three is shown; (**b**) densitometric quantification of the PDX-1 protein after normalization to the loading control at each time point. Mean ± S.D. stands for three independent experiments; (**c**) MIN6 cells transiently transfected with plasmids encoding FLAG-PDX-1_WT_ or FLAG-PDX-1_S232A/T231A_ were treated 48 h post transfection with (+) or without (−) 5 µM MG132 for 11 h. Cells were then lysed and the whole cell extracts were analyzed by Western blotting using anti FLAG-tag and anti-α-tubulin antibodies. α-tubulin was analyzed as a loading control. A quantification of the relative FLAG-PDX-1 protein level (FLAG-PDX-1/α-tubulin) is shown in (**d**). The results were the mean ± S.D. of three independent experiments.

In the next step we asked whether binding of PDX-1 to PCIF1 might have an influence on the stability of PDX-1. To determine the stability of PDX-1 wild-type, as well as of the CK2 phosphorylation mutant PDX-1_T231A/S232A_, MIN6 cells were transfected with PDX-1 constructs and 30 h after transfection cells were treated with cycloheximide for an inhibition of protein synthesis. Cells were either extracted immediately (0 h) or after 3, 6, 12, or 24 h of treatment. The cell extracts were analyzed on an SDS polyacrylamide gel followed by Western blot with an anti-FLAG-tag-antibody. As shown in Figure 4a, over a time course of 24 h, wild-type PDX-1 degraded more rapidly than the mutant PDX-1_T231A/S232A_. The experiment was done in triplicate, the resulting bands for PDX-1 were scanned, quantified and normalized to the corresponding α-tubulin loading control. The mean ratios (PDX-1/ α-tubulin) +/- standard deviation of the densitometric quantification are shown in Figure 4b. The calculated half-life of wild-type PDX-1 was 12 h, which is in the range of published data [28,29] whereas, the half-life of mutant PDX-1_T231A/S232A_ was about 24 h. Thus, PDX-1, which can no longer be phosphorylated by CK2, is much more stable than the corresponding wild-type form.

It was shown that PCIF1 and Cullin 3 target PDX-1 for ubiquitination [21]. Therefore, we next tested whether the CK2 phosphorylation of PDX-1 might have an influence on its proteasomal degradation. MIN6 cells were transfected with FLAG-tagged wild-type PDX-1 or with mutant PDX-1_S232A/T231A_ for 48 h. Cells were treated with or without 5 µM MG132, a proteasome inhibitor, for another 11 h and lysed. PDX-1 protein levels were analyzed on a 12.5% SDS polyacrylamide gel and transferred to a PVDF membrane followed by Western blotting using anti-FLAG tag and anti-α-tubulin antibodies.

As shown in Figure 4c, treatment with MG132 led to a distinct accumulation of wild-type PDX-1 protein, whereas no significant change was observed in the PDX-1 double alanine mutant protein level. After quantification of the expression of both forms from three independent experiments the increase for the wild-type protein turned out to be more than 50% (Figure 4d). This observation meant that the phosphorylation mutant was less sensitive than wild-type PDX-1 to MG132 treatment, which indicated that the non-phosphorylated form was more resistant to proteasomal degradation whereas wild-type PDX-1 was targeted for degradation by the proteasome machinery.

From these various results we conclude that CK2 phosphorylation of PDX-1 has a massive influence on the stability of PDX-1 and this phosphorylation confers susceptibility of PDX-1 protein to proteasomal degradation.

## 3. Discussion

**Figure 5:**
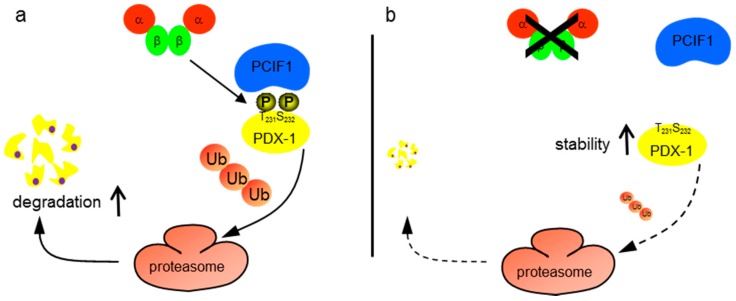
Model of the PDX-1-PCIF1 interaction (**a**) CK2 phosphorylates mouse PDX-1 at threonine 231 and serine 232. This site is located in the middle of a phosphodegron, which binds the E3 ubiquitin ligase protein PCIF1 in a phosphorylated form. PCIF1 targets PDX-1 to the proteasome where it is ubiquitinated and subsequently degraded; and (**b**) upon CK2 inhibition, T231 and S232 of PDX-1 are not phosphorylated. As these important residues of the phosphodegron are not phosphorylated, the interaction with PCIF1 is reduced and PDX-1 is more stable.

Protein kinase CK2 is classified as a serine/threonine kinase with more than 400 different substrates, which indicates the pleiotropic functions of CK2 [4]. We have recently identified PDX-1 as a substrate of CK2. PDX-1 is phosphorylated by CK2 at threonine 231 and serine 232 in the C-terminus [11]. By using CK2 inhibitors as well as by using PDX-1 where the threonine and the serine residues were mutated to alanine, it was shown that the CK2 phosphorylation of PDX-1 regulates its transcription factor activity. Here, we show that CK2 phosphorylation of PDX-1 regulates its stability. Furthermore, we demonstrated that CK2 phosphorylation influences the binding of PDX-1 and PCIF1, a factor which targets PDX-1 for proteasomal degradation. Our observations are summarized in Figure 5.

The stability of some proteins, such as myc [7], ataxin-3 [30], SAG-SCFE3 ubiquitin ligase [31], PML [8], PTEN [9], VHL [10], and NKX3.1 [32], is regulated by CK2 phosphorylation. Here, we show that the use of PDX-1, which could no more be phosphorylated by CK2 resulted in an elevated stability of PDX-1. There are some other reports showing that the stability of PDX-1 is regulated by phosphorylation. PDX-1 is phosphorylated in vivo on serine 61 and/or serine 66 by glycogen synthase kinase 3 (GSK3) in pancreatic β-cells [29]. This phosphorylation targets the protein for degradation by the proteasome, which results in a decreased half-life for the PDX-1 protein. In contrast, treatment of cells with a proteasome inhibitor led to distinct accumulation of GSK3 phosphorylated PDX-1 protein at serine 61/serine 66, whereas no significant change was observed for total PDX-1 protein. PDX-1 is also phosphorylated by the DNA-dependent protein kinase (DNA PK) and by the mammalian sterile 20-like kinase-1 (MST1) in the N-terminus at threonine 11 [33,34]. This phosphorylation resulted in the ubiquitination and degradation of PDX-1. It is of interest that under diabetic conditions, MST1 was strongly activated in β-cells of human and mouse islets. An et al. [35] recently reported that murine PDX-1 is phosphorylated at serine 269 by the homeodomain interacting protein kinase 2 (HIPK2). Treatment of β-cells with high glucose concentrations resulted in an enhanced phosphorylation at serine 269. It was further shown that HIPK2 phosphorylation at this site affected neither the stability of PDX-1 nor its transactivation potential. However, this phosphorylation affected the subcellular distribution of PDX-1 in MIN6 cells. The same site is also target of p38 MAPK phosphorylation [36]. p38 interacted with PDX-1 and phosphorylated the human protein at serine 268, which resulted in an increased expression and decreased ubiquitination of PDX-1. In βTC3 insulinoma cells this phosphorylation is induced upon a stimulus with glucagon like peptide GLP1 and acts as a positive regulator of PDX-1. In line with these observations we found that the PDX-1 stability was also affected by the CK2 phosphorylation of PDX-1 at threonine 231 and serine 232.

For the ubiquitination and subsequent proteasomal degradation of proteins a highly controlled orchestra of activating E1 enzymes, E2 conjugating enzymes and E3 ubiquitin ligase enzymes is necessary [36]. Targeting the substrates for ubiquitin-mediated degradation is the responsibility of adaptor proteins. Murine PCIF1, or its 100% human homologue SPOP, was identified as such an adaptor protein, which bridges the Cullin 3 moiety of an E3 ligase and its substrate [22]; PDX-1 was identified as one of these PCIF1-binding substrates [20]. Zhuang et al. [37] published a characteristic PCIF1 binding consensus motif which consists of Π-π-S-S/T-S/T (Π = nonpolar, π = polar). In SRC-3, another PCIF1-binding substrate, the binding region was defined as DVSST, where the polar and nonpolar residues occur in a reversed order [38]. For the interaction of PDX-1 and PCIF1, amino acids 210–238 of mouse PDX-1 are essential amino acids [20]. When checking this region (RSSGTPSGGGGGEEPEQDCAVTSGEELLA), we noticed the sequence _229_AVTS_232_ which is quite similar to the SRC-3 binding motif of PCIF1. The threonine and serine residue within this consensus motif are the phosphorylation sites for protein kinase CK2. We found that the binding of PDX-1 to PCIF1 was enhanced upon using phospho-mimicking mutants where T231 and S232 were exchanged by an acid residue; on the other hand, PDX-1 binds weaker to PCIF1 upon inhibition of CK2. There are several examples that binding of E3 ubiquitin ligases, like β-TRCP or FBW7, to their substrates is modulated by phosphorylation [39,40,41]. So-called phosphodegrons were also found for the PCIF1 substrates SRC-3 [38] and the ERG oncoprotein [42]; in both cases serine residues within the consensus motif are phosphorylated by CK1. The phosphorylation triggers the interaction between the E3 ligase adaptor protein PCIF1 and its substrate and thereby activates degradation.

PCIF1 is a nuclear protein with a speckled pattern of nuclear staining [20], PDX-1 and PCIF1 co-localize in the nucleus [20] and our observation). PCIF1 together with Cullin 3 mediates the ubiquitination of PDX-1 which was shown in ubiquitin overexpression systems [20,43]. Here, we have shown the influence of CK2 phosphorylation of PDX-1 upon its ubiquitination and proteasomal degradation by using the proteasome inhibitor MG132 [44]. We observed that the phosphorylation mutant of PDX-1 had a significant prolonged half-life (24 h) compared with the wild-type PDX-1 (12 h). Furthermore, the wild-type PDX-1, rather than the mutant form of PDX-1, accumulated in response to treatment with the proteasome inhibitor MG132 in β-cells. Thus, all these findings let us conclude that CK2-phosphorylated PDX-1 underwent rapid degradation in β- cells by the proteasomal pathway, further supporting the idea that modification by CK2 phosphorylation served as an important part to regulate the stability of the PDX-1 protein.

Some studies suggested a stimulatory effect of glucose on PDX-1 phosphorylation [16,22,32], whereas others showed that glucose stimulation of β-cells decreases PDX-1 phosphorylation [15]. Moreover, a decrease in C-terminal phosphorylation of PDX-1 was associated with an increase in protein stability. Here, we demonstrated a decrease in binding of PDX-1 to PCIF1 upon CK2 inhibition, which may be the reason for the enhanced stability of the phospho-deficient alanine mutant. The significance of phosphorylation of PDX-1 for the interaction is more obvious under high glucose conditions, a situation where the presence of a fully functional PDX-1 is extremely important for the regulation of insulin transcription, secretion and regulation of blood glucose homeostasis. Therefore, inhibition of CK2 seems to be an option to increase the stability of PDX-1 and subsequently, the level of insulin. A CK2 inhibitor is currently under clinical investigation for the treatment of cancer [24]. It may be an interesting question whether this inhibitor can also be used to treat *Diabetes mellitus*.

## 4. Materials and Methods

### 4.1. Cell Culture and Treatment

The mouse cell line MIN6 [45] was maintained in Dulbecco’s modified Eagle’s medium (Sigma-Aldrich, Munich, Germany) containing 5.5 mM d-glucose and 2 mM glutamine supplemented with 15% (*v*/i) fetal bovine serum and 100 μM β-mercaptoethanol in humidified 5% CO_2_/95% air at 37 °C. 

The CK2 inhibitor CX-4945 (SelleckChem, Munich, Germany) was dissolved in dimethyl sulfoxide (DMSO) to a 10 mM stock solution, which was used to treat the cells with the given final concentration. In control experiments we used the same volume of the solvent DMSO alone. To check the stability of the PDX-1 protein, MIN6 cells were transfected with FLAG-tagged PDX-1_WT_ or with mutant PDX-1_T231A/S232A_ and then further incubated for 30 h. The proteasome inhibitor MG132 (Calbiochem, Darmstadt, Germany) was dissolved as 10 mM stock solution in DMSO and applied on transfected cells 48 h post transfection in a final concentration of 5 µM for 11 h.

### 4.2. Plasmids

Full-length mouse PDX-1 was subcloned into the mammalian expression vector p3xFLAG/CMV-7.1 (Sigma-Aldrich, Munich, Germany) using the BamH1 site. PDX-1 phosphorylation mutants were generated using a QuikChange site-directed mutagenesis kit (Stratagene, La Jolla, CA, USA) according to the manufacturer’s protocol. As another eukaryotic expression vector we used pcDNA3.1-PDX-1_WT_ [11]. PDX-1_WT_ and mutants were also ligated with the bacterial expression vector pGEX-4T-1 (GE Healthcare, Freiburg, Germany) via its BamH1 site [11]. For mammalian expression of human PCIF1 we used pFLAG-CMV-2-SPOP, a kind gift of C. H. Chung, Seoul, South Korea [46]. The cDNA of human PCIF1 was also cloned into the BamH1/Xho1 sites of pET28a. All constructs generated by cloning and mutagenesis were verified by DNA sequencing.

### 4.3. Extraction of Cells and Western Blot Analysis

For harvesting, cells were scraped off the plate and sedimented by centrifugation (7 min, 4 °C, 400× *g*). Cells were washed with cold phosphate-buffered saline (PBS) and lysed with the double volume of RIPA buffer (50 mM Tris/HCl, pH 8.0, 150 mM NaCl, 0.5% sodium desoxycholate, 1% Triton X-100, 0.1% sodium dodecylsulfate) supplemented with the protease inhibitor cocktail complete^TM^ (Roche Diagnostics, Mannheim, Germany). After lysis, cell debris was removed by centrifugation. The protein content was determined according to a modified Bradford method (BioRad, Munich, Germany). SDS polyacrylamide gel electrophoresis and Western blot analysis was performed essentially as described [11]. PDX-1 was detected using a polyclonal antiserum from rabbit. The mouse monoclonal antibody FLAG M2 (F1804) and the mouse monoclonal antibody β-tubulin (clone DM1A) were from Sigma-Aldrich (Munich, Germany).

### 4.4. Co-Immunoprecipitation

For co-immunoprecipitation experiments, MIN6 cells were transfected with pcDNA3.1-PDX-1 and pFLAG-CMV-2-SPOP using Effectene™ according to the provider (Qiagen, Hilden, Germany). 24 h after transfection, cells were extracted and 4 mg of total protein was subjected to immunoprecipitation. The cell lysates were pre-cleared twice with a mixture of protein A sepharose beads and CL 4-B sepharose beads (GE Healthcare, Freiburg, Germany) over a period of 1 h. The supernatant was incubated with a rabbit anti PDX-1 antibody for 2 h. Beads were washed four times with PBS (137 mM NaCl, 2.7 mM KCl, 8 mM Na_2_HPO_4_, 1.5 mM KH_2_PO_4_, pH 7.4). Bound proteins were eluted with SDS sample buffer (130 mM Tris-HCl, pH 6.8, 0.02% bromophenol blue (*w*/*v*), 10% β-mercaptoethanol, 20% glycerol (*v*/*v*), and 4% SDS), and analyzed by Western blot with the M2 FLAG-antibody for PCIF1 and the polyclonal rabbit antiserum against PDX-1. Proteins were visualized by the ECL Lumilight system of Roche Diagnostics (Mannheim, Germany).

### 4.5. Cycloheximide Chase 

MIN6 cells were either transfected with FLAG-PDX-1_wt_ or FLAG-PDX-1_T231A/S232A_ mutant using Effectene™ (Qiagen, Hilden, Germany) as recommended by the manufacturer. Thirty hours after transfection, MIN6 cells were cultured in growth medium containing 100 µg/mL cycloheximide (Sigma-Aldrich GmbH, Munich, Germany) for 0, 3, 6, 12, or 24 h. Cells were harvested and lysed with RIPA buffer as described earlier and extracts were subjected to SDS polyacrylamide gel electrophoresis and Western blot analysis. The experiment was performed in triplicate. The PDX-1 signals were scanned and densitometrically quantified using the Quantity One (version 4.6.7.) software from BioRad (Munich, Germany). The values were normalized to an α-tubulin loading control which was analyzed on the same gel. The mean values +/- standard deviation of three independent experiments were represented in the corresponding graphs. 

### 4.6. Immunofluorescence Analysis

MIN6 cells were seeded in a 6 cm plate on coverslips, cultured overnight, and immunofluorescence analysis was performed as described [47]. For identification of the PDX-1 protein, a rabbit polyclonal antiserum generated against the C-terminus of PDX-1 was used. PCIF1 was detected with a goat polyclonal antibody (SPOP K-13 from Santa Cruz Biotechnologies, Heidelberg, Germany). Secondary antibodies were goat anti rabbit AlexaFluor^TM^594 and donkey anti goat AlexaFluor^TM^488 (Invitrogen, Karlsruhe, Germany). Nuclei were visualized by 4,6-diamino-2-phenylindole (DAPI) staining. Analysis was carried out by fluorescence imaging performed on an Axioskop fluorescence microscope (Zeiss, Jena, Germany).

### 4.7. Duolink^®^ in Situ Proximity Ligation Assay

For detection of the interaction between the PDX-1 protein and PCIF1, we used the Duolink^®^ in situ Proximity Ligation Assay (PLA, Sigma-Aldrich GmbH, Munich, Germany) according to the manufacturer’s protocol with MIN6 cells. PDX-1 was detected with the polyclonal rabbit antiserum, PCIF1 with a mouse monoclonal antibody (SPOP B-8, Santa Cruz Biotechnologies, Heidelberg, Germany). Detection of the interaction signals was carried out by fluorescence imaging performed on an Axioskop fluorescence microscope (Zeiss, Jena, Germany).

### 4.8. GST-Pull Down Assay

The pull-down assay was essentially done as described by Sun et al. [48]. Purified GST-tagged proteins (10 μg) were immobilized on GSH-sepharose and equilibrated with PBS-T binding buffer (PBS, pH 7.4, 1% Tween 20). Immobilized proteins were incubated for 2 h at 4 °C with 7.5 µL of PCIF1 product from an in vitro translation reaction. The translation from a pET28a-PCIF-1 plasmid was done as recommended by the manufacturer (Promega GmbH, Mannheim, Germany). After washing with cold PBS-T, bound proteins were eluted with SDS sample buffer (65 mM Tris-HCl, pH 6.8, 2% SDS, 5% β-mercaptoethanol, 10% glycerol, 0.01% bromophenol blue) and analyzed by SDS polyacrylamide gel electrophoresis, followed by protein staining with Coomassie blue and autoradiography.

### 4.9. In Vitro Phosphorylation

To determine the activity of CK2 after its inhibition, cells were treated with CX-4945 or left untreated, lysed, and the extracts were used in a kinase filter assay. In this assay we measured the incorporation rate of [^32^P]-phosphate into the synthetic CK2 specific substrate peptide with the sequence RRRDDDSDDD [14]. Twenty microliter kinase buffer (50 mM Tris/HCl, pH 7.5, 100 mM NaCl, 10 mM MgCl_2_, 1 mM dithiotreitol (DTT)) containing 30 µg protein were mixed with 30 µL CK2 mix (25 mM Tris/HCl, pH 8.5, 150 mM NaCl, 5 mM MgCl_2_, 1 mM DTT, 50 μM ATP, 0.19 mM substrate peptide) containing 10 μCi/500 μl [^32^P]γ ATP. The mixture was spotted onto a P81 ion exchange paper. The paper was washed three times with 85 mM H_3_PO_4_. After treatment with ethanol, the paper was dried and the Čerenkov radiation was determined in a scintillation counter.
